# Nomogram to Predict Cognitive State Improvement after Deep Brain Stimulation for Parkinson’s Disease

**DOI:** 10.3390/brainsci12060759

**Published:** 2022-06-09

**Authors:** Bowen Chang, Chen Ni, Weiwen Zhang, Jiaming Mei, Chi Xiong, Peng Chen, Manli Jiang, Chaoshi Niu

**Affiliations:** 1 Department of Neurosurgery, The First Affiliated Hospital of USTC, Division of Life Sciences and Medicine, University of Science and Technology of China, Hefei 230001, China; changbowen21@163.com (B.C.); chenni_edu@126.com (C.N.); 18130619327@163.com (W.Z.); doctormeijiaming@163.com (J.M.); xc0624010052@126.com (C.X.); 15055163720@163.com (P.C.); dyjiangmanli@126.com (M.J.); 2 Anhui Province Key Laboratory of Brain Function and Brain Disease, Hefei 230001, China

**Keywords:** deep brain stimulation, Parkinson’s disease, cognitive state, non-motor symptoms, nomogram

## Abstract

Purpose: Parkinson’s disease (PD) is a common neurodegenerative disease, for which cognitive impairment is a non-motor symptom (NMS). Bilateral subthalamic nucleus deep brain stimulation (STN-DBS) is an effective treatment for PD. This study established a nomogram to predict cognitive improvement rate after STN-DBS in PD patients. Methods: We retrospectively analyzed 103 PD patients who underwent STN-DBS. Patients were followed up to measure improvement in MoCA scores one year after surgery. Univariate and multivariate logistic regression analyses were used to identify factors affecting improvement in cognitive status. A nomogram was developed to predict this factor. The discrimination and fitting performance were evaluated by receiver operating characteristics (ROC) analysis, calibration diagram, and decision curve analysis (DCA). Results: Among 103 patients, the mean improvement rate of the MoCA score was 37.3% and the median improvement rate was 27.3%, of which 64% improved cognition, 27% worsened cognition, and 8.7% remained unchanged. Logistic multivariate regression analysis showed that years of education, UPDRSIII drug use, MoCA Preop, and MMSE Preop scores were independent factors affecting the cognitive improvement rate. A nomogram model was subsequently developed. The C-index of the nomogram was 0.98 (95%CI, 0.97–1.00), and the area under the ROC was 0.98 (95%CI 0.97–1.00). The calibration plot and DCA demonstrated the goodness-of-fit between nomogram predictions and actual observations. Conclusion: Our nomogram could effectively predict the possibility of achieving good cognitive improvement one year after STN-DBS in patients with PD. This model has value in judging the expected cognitive improvement of patients with PD undergoing STN-DBS.

## 1. Introduction

Parkinson’s disease (PD) typically develops between the ages of 55 and 65 years, affecting 1% to 2% of people over 60 years and approximately 0.3% of the general population, with a higher prevalence in men than in women [1,2]. The main motor symptoms of PD include resting tremors, bradykinesia, and rigidity. Patients also experience a variety of non-motor symptoms (NMS), including mood disorders (e.g., depression and anxiety), cognitive disorders (e.g., frontal lobe dysfunction, memory difficulties, and dementia), sleep disorders (e.g., apnea, sleep disorders, and RDB), and autonomic nervous dysfunction (e.g., sexual dysfunction, sleep disorders, constipation, and bladder symptoms) [3]. Since Prof. Benabid first applied deep brain stimulation (DBS) to the treatment of movement disorders in 1987, it has experienced more than 30 years of development [4]. DBS has gradually become one of the most effective treatments for patients with advanced PD. Many previous studies have shown that DBS can significantly improve the motor symptoms of PD patients, but whether DBS can improve the cognitive status of patients remains controversial [5]. Similarly, there is a lack of clinical models to predict improvements in the cognitive status of patients with PD using DBS. The development of a nomogram could remedy this deficiency by integrating multiple important factors to model and predict whether PD patients can achieve better cognitive improvement after DBS. Here, we conducted a retrospective study of bilateral subthalamic nucleus DBS(STN-DBS) in PD patients in our center, aiming to develop and verify a nomogram for PD patients after STN-DBS, and to predict whether patients could achieve better cognitive status improvement after one year.

## 2. Materials and Methods

### 2.1. Patients

From September 2019 to April 2021, all medical records and questionnaire results were retrospectively obtained from patients diagnosed with PD undergoing STN-DBS at The First Affiliated Hospital of USTC. The Institutional Ethics Committee of our hospital approved the study protocol. 

### 2.2. Outcome Assessment

Demographic and clinicopathological variables of the enrolled patients were collected from their medical records and questionnaires, including age, sex, duration, and levodopa equivalent dose (LED). Patients were assessed using a detailed scale to assess the severity of their symptoms, cognitive state, psychological status, and quality of life. The UPDRS-III was used to assess the severity of the patient’s symptoms. The NMSS (Parkinson s Nonmotor Symptom Scale) assesses Parkinson’s nonmotor symptoms. The PDQ-39 scale was used to evaluate quality of life. The Hamilton Anxiety (HAMA) and Depression (HAMD) Scale was used to evaluate the psychological state of the patients. The Montreal Cognitive Assessment (MoCA) and Mini-Mental State Examination (MMSE) scales were used to evaluate cognitive state. One year later, the patients’ cognitive status was assessed again using the MoCA scale. PD patients were divided into two groups according to the median improvement rate of the MoCA score one year after surgery. Patients with MoCA score improvement rates above the median were classified into the high improvement rate group, and those with MoCA score improvement rates below the median were classified into the low improvement rate group.

### 2.3. Statistical Analyses

Empower (R) (www.empowerstats.com, X&Y Solutions, Inc., Boston, MA, USA; accessed on 1 January 2022), together with R (http://www.R-project.org; accessed on 1 January 2022), was employed for all statistical analyses. Initially, the normal distribution of the variables was examined using the Kolmogorov–Smirnov test. Data conforming to a normal distribution were evaluated using a two-tailed Student’s *t*-test, one-way analysis of variance (ANOVA), and a Shapiro–Wilk test. Non-parametric data were compared among different groups using the Mann–Whitney U test. Logistic regression multivariate analysis of variance was used to explore related risk factors. Subsequently, these risk factors were used to develop the regression models. These models were then transformed into a nomogram. Calibration plots, receiver-operating characteristic curves, and decision curve analysis diagrams were used to assess the models. Correlations of the MoCA score improvement rate with MoCA preoperative, education, MMSE preoperative, and UPDRSIII drug were analyzed using Pearson’s correlation coefficient test. 

## 3. Results

### 3.1. Patients

**Figure 1:**
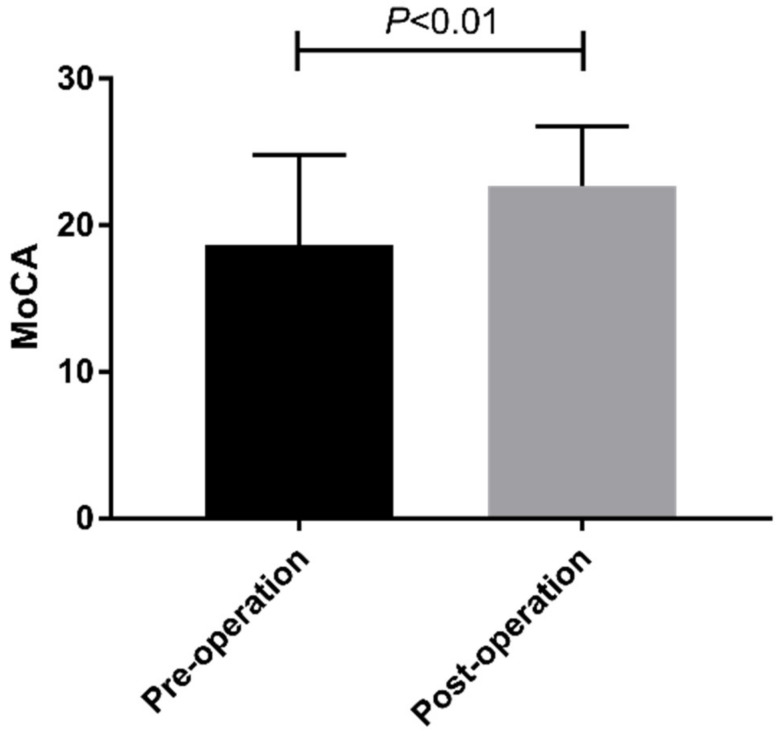
Comparison of MoCA score between pre-operation and one year after operation.

**Figure 2:**
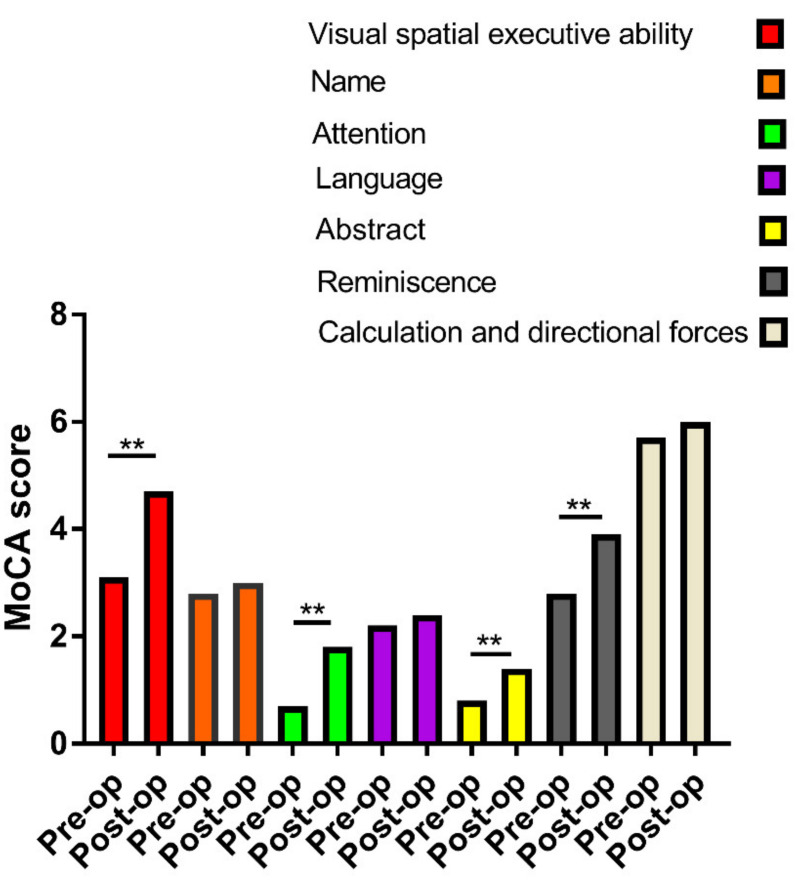
Comparison of details of MoCA score between preoperative and postoperative one year. ** *p* < 0.01.

The final analysis included 103 PD patients. All patients were enrolled consecutively, and none of the 103 patients had been lost to follow-up. The demographic data of all the subjects are shown in Table 1. The enrolled PD patients were 35–75 years old, comprising 63 men (61.17%) and 40 women (38.83%). One year after STN-DBS, the mean improvement rate in the MoCA score was 37.3%, and the median improvement rate was 27.3%. Compared with preoperative values, STN-DBS could significantly improve patients’ MoCA scores (Figure 1). Cognitive status improved in 64% of the patients, worsened in 27%, and remained unchanged in 8.7%. We divided the patients into high and low improvement groups based on the median improvement rates. At one year after STN-DBS, patients’ MoCA scores showed significant improvements in visual and spatial executive ability, attention, abstract thinking, and reminiscence (Figure 2).

**Figure 3:**
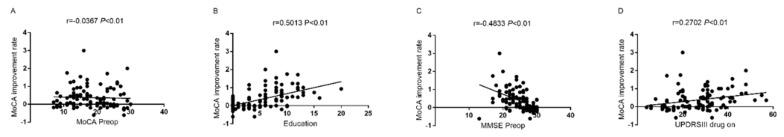
Correlations of MoCA score improvement rate with MoCA preop (**A**), education (**B**), MMSE preop (**C**) and UPDRSIII drug on (**D**).

The group with a high improvement rate comprised 22 women (42.31%) and 30 men (57.69%), with an average age of 60.67 years. In the lower improvement rate group, there were 18 women (35.29%) and 33 men (64.71%), with an average age of 57.31 years. There were significant differences in patient age, education, drug improvement rate, UPDRSIII drug on, UPDRSIII drug off, MoCA Preop, and MMSE Preop between the two groups. It is worth noting that MoCA Preop data is 23.76 ± 3.44 for the low improvement rate and 13.67 ± 3.57 for the high improvement rate. These could be really skewed base data points. Table 2 shows the results of univariate analysis for each variable. The results showed that the degree of improvement in the MoCA scores correlated with age, education, drug improvement rate, UPDRSIII drug on, UPDRSIII drug off, MoCA Preop, and MMSE Preop. Table 3 shows the multiple regression analysis results of variables in the higher and lower improvement rate groups. Multivariate regression analysis showed that education (OR = 1.49, 95%CI: 1.25–1.77, *p* < 0.001), UPDRSIII drug use (OR = 1.08, 95%CI: 1.02–1.14, *p* < 0.01), MoCA Preop (OR = 0.14, 95%CI: 0.04–0.54, *p* < 0.01), and MMSE Preop(OR = 0.61, 95%CI: 0.49–0.77, *p* < 0.001) were independent risk factors affecting the improvement of MoCA score in PD patients after STN-DBS. In addition, MoCA Preop and MMSE Preop scores of PD patients were negatively correlated with the improvement rate of the MoCA score one year after surgery. In contrast, years of education and UPDRSIII drug on the score of PD patients were positively correlated with the improvement rate of the MoCA score one year after surgery (Figure 3).

### 3.2. Development of the Nomogram 

**Figure 4:**
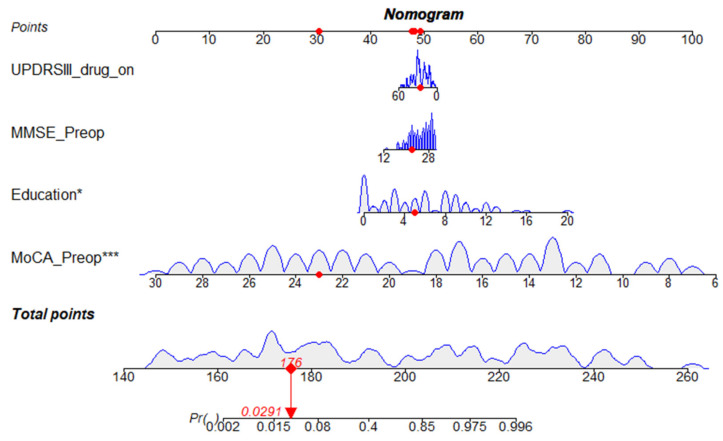
Nomogram to predict improvement of MoCA score after STN-DBS for PD. Clinical factor corresponds to a specific point by drawing a line straight upward to the points axis. After the sum of the points is located on the total points axis, the sum represents the probability of obtaining higher a MoCA score improvement rate. * *p* < 0.01, *** *p* < 0.001.

**Figure 5:**
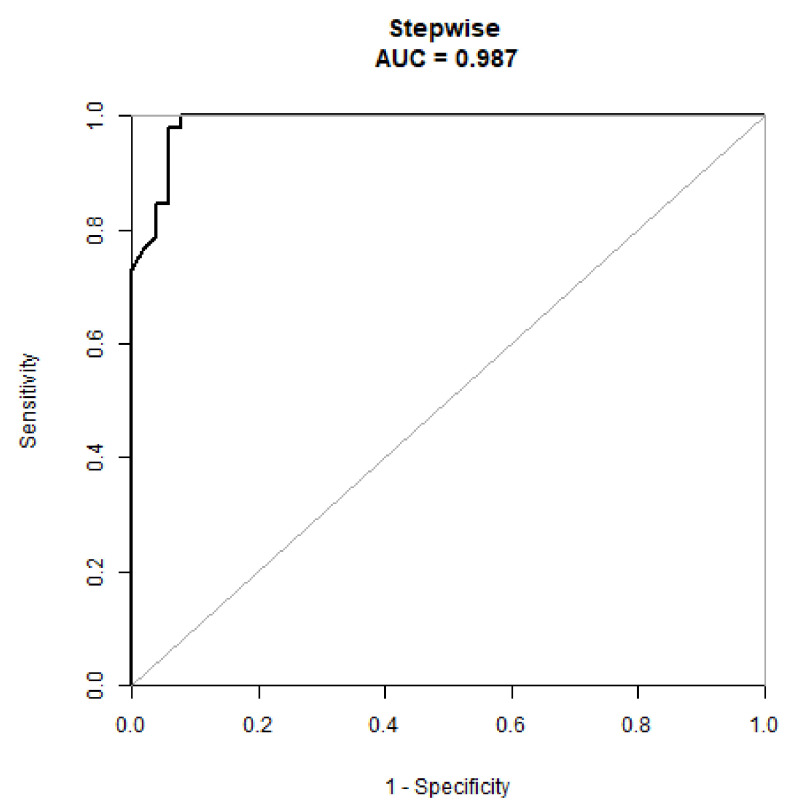
A receiver operating characteristic curve to evaluate the discriminating capability of the nomogram.

Based on these results, we developed a prediction model and generated a line graph to predict whether the MoCA score could be improved one year after STN-DBS surgery (Figure 4). Each clinical factor corresponds to a specific score, and a linear point axis was plotted to calculate the total score, which corresponds to a higher probability of MoCA score improvement. As can be seen from Figure 2, the prediction model has good discriminant ability, and the area under the ROC curve is 0.98 (95%CI, 0.97–1.00) (Figure 5). The C-index of the model with a better MoCA score was 0.98 (95%CI, 0.97–1.00).

### 3.3. Validation of the Nomogram

**Figure 6:**
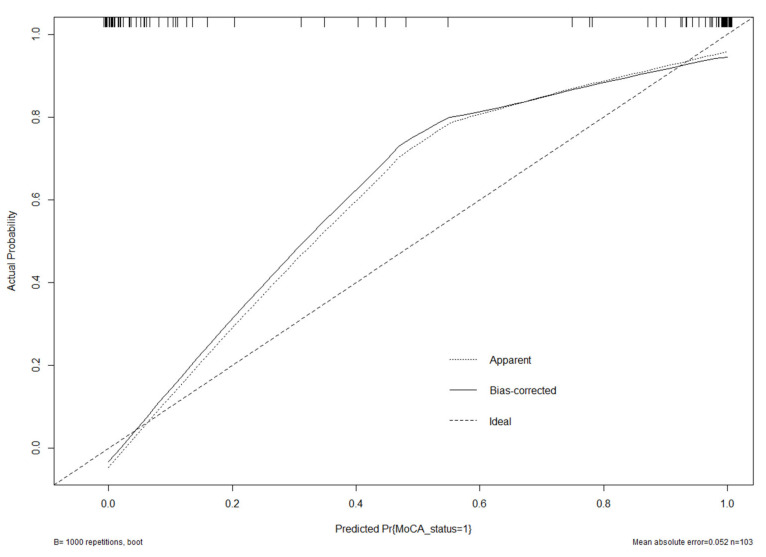
Calibration curve of the model. The calibration of the model in line with the agreement between predicted and observed outcomes of improvement of MoCA score.

**Figure 7:**
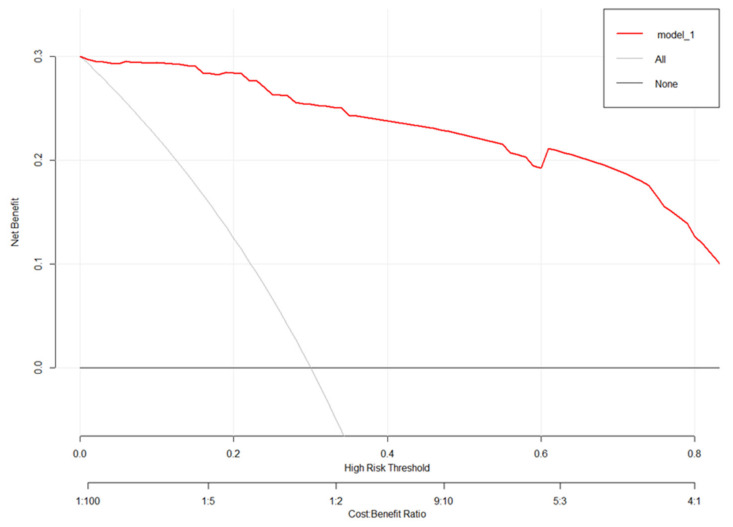
The decision curve analysis diagram of the model.

The generated model was verified internally using the bootstrap verification method, and the C-index of the bootstrap correction was 0.98. In addition, calibration curves were generated by plotting the actual and predicted improvements in MoCA scores between the better MoCA score improvements (*Y*-axis) and better MoCA score improvements (*X*-axis). The results in Figure 6 show good agreement between the predictions and observations. In addition, the decision curve analysis (DCA) was drawn with the net benefit rate as the ordinate, and the high-risk threshold as the abscissa (Figure 7), where the high-risk threshold was set to (0.1). As shown in Figure 7, when the high-risk threshold was 0–1, the net benefit rate was greater than 0, which was clinically significant.

## 4. Discussion

The use of DBS for the treatment of NMS in patients with PD has gradually become a hotspot in recent studies. NMS includes behavioral and mental changes, cognitive changes, autonomic nervous system failure, sensory disturbances, and sleep disturbances [6,7,8]. However, compared with motor symptoms, improvements in NMS, especially in cognitive status, are often overlooked and poorly managed by clinicians [9]. A total of 103 patients with PD were included in this study to analyze the related factors that may affect whether they can obtain a good cognitive state of improvement one year after STN-DBS. Patients with PD typically show varying degrees of cognitive impairment, with mild cognitive impairment (MCI) occurring in 25% of patients in a large cohort study, and dementia occurring in 20–70% of patients in a series of other studies [10,11]. A recent meta-analysis, which included all DBS treatment targets from PD to essential tremor (ET) and dystonia, found that cognitive function improved by 31%, deteriorated by 12%, and remained unchanged by 13% [12]. However, other studies have shown that STN-DBS decreases overall cognition, memory, verbal fluency, and executive function [13,14]. In our cohort, we found that STN-DBS effectively improved the cognitive status of the patients. One year after surgery, 64% of the patients showed an improved cognitive status, 27% had deteriorated, and 8.7% remained unchanged. Based on the above, it is important to explore the factors that affect the benefit of patients’ cognitive state improvement after STN-DBS. 

In a previous cohort study, 36% of surgical patients experienced cognitive decline one year after surgery. Predictors of cognitive decline include preoperative executive dysfunction, advanced age, and poor dopamine responses in the left atrium [15]. This study found that the improvement in patients’ cognitive status one year after surgery may be related to their age, years of education, drug improvement rate, UPDRSIII drug on, UPDRSIII drug off, MoCA Preop, and MMSE Preop. These factors were further investigated in the present study. Through multivariate logistic regression analysis, we found that the patients’ years of education, UPDRSIII drug use, MoCA Preop, and MMSE Preop were independent factors influencing improvement in cognitive status one year after surgery. Education is an important means of improving the human cognitive level [16]. Years of education have been widely reported to be positively correlated with cognitive status [17,18]. Our study showed that patients with more years of education were more likely to achieve satisfactory cognitive improvement one year after surgery. In addition, there was a negative correlation between the MoCA score improvement rate and preoperative MoCA and MMSE scores, which were also independent factors affecting patients’ achievement of better cognitive improvement. Patients with more severe preoperative cognitive impairment had a greater chance of improvement, and thus, may have a higher rate of improvement. Therefore, the degree of cognitive impairment in patients should be fully evaluated before surgery to obtain a reasonable expectation of improvement in cognitive state after treatment. Concurrently, the preoperative UPDRSIII drug score was positively correlated with the improvement rate of the MoCA score, which was also an independent factor affecting postoperative cognitive improvement. This suggests that the more severe the motor symptoms before surgery, the more likely it is to achieve better cognitive improvement after surgery.

The exact mechanisms of cognitive changes after STN DBS still stay unclear. Sophisticated fiber connections exist between STN and cognition-related regions such as limbic and association areas, which might be influenced by chronic stimulation. STN provides the only excitatory fiber to basal ganglia, part of cognition-related basal ganglia-thalamusdorsal prefrontal cortex circuitry [19]. Electrical stimulation at the site of STN could result in changes in this circuitry as is shown in a study of 10 patients revealing intraoperative blood oxygen level-dependent (BOLD) signal changes in both motor and limbic circuitry [20].

However, even if patients had the same risk factors, the likelihood of achieving better cognitive improvement was quite different. Poor cognitive improvement is caused by multiple factors, while single-factor analysis may unilaterally affect prognosis, often missing other important factors, thus losing accurate judgment of the prognosis of patients. A nomogram is a useful tool to predict clinical outcomes of patients and can integrate the influence of various factors on patient outcomes. It has been widely used in the survival analysis of cancer patients and has gradually replaced the traditional prediction model. Unfortunately, this predictive model is rarely used for patients with PD who underwent DBS. So far, only Wang et al. developed a nomogram based on postoperative delirium in PD patients after STN-DBS in 2019, and Frizon LA developed a nomogram based on postoperative quality of life improvement in PD patients after DBS in 2019 [21,22,23]. No studies have yet reported cognitive improvement one year after STN-DBS. Herein, we incorporated 103 patients treated for PD from 2017 to 2020, into a model to construct the first nomogram model of postoperative cognitive state improvement in patients with PD undergoing STN-DBS. Through multivariate analysis, four factors, including years of education, MoCA Preop, MMSE Preop, and UPDRSIII drugs were used as nomogram scores. The model was found to have a good predictive ability. The C index was 0.98 (95%CI, 0.97–1.00). The calibration graph in the validation queue represents an exact case.

This study had several limitations. First, the single-center design will have introduced inherent selection bias. Further, we were unable to clarify whether race, diet, climate, or other factors influence the likelihood that patients will achieve better cognitive improvement. Whether this model can be used in primary hospitals or in other areas has not yet been proven. In the future, we hope to conduct further multi-center studies which would allow us to randomly select patients with PD from other centers for external validation. Furthermore, as this was a retrospective cohort study, bias in the follow-up process was inevitable. In addition, due to the lack of data in this study, we neglected the analysis of motor symptoms/frozen gait. Of course, what is more important is that the follow-up time of this study is too short. In a later study, we will provide long-term follow-up results to explore the influence of STN-DBS on patients’ cognition.

## 5. Conclusions

In conclusion, we developed a nomogram to predict the possibility of achieving good cognitive improvement one year after STN-DBS in patients with PD. Analysis of the ROC curve, calibration plot, and DCA curve showed that the nomogram had good predictive performance and calibration. This model has value in judging the expected cognitive improvement of patients with PD treated with STN-DBS.

## Figures and Tables

**Table 1:** Comparison between patients with higher improvement rate of cognitive state and those with lower rate of improvement.

	Lower Improvement Rate	Higher Improvement Rate	*p*-Value
No.	51	52	
Age	57.31 ± 7.95	60.67 ± 7.82	0.033
Duration (years)	8.86 ± 3.95	8.54 ± 3.67	0.667
Education (years)	3.13 ± 2.95	7.90 ± 4.29	<0.001
LED	661.03 ± 215.73	658.89 ± 468.04	0.976
Drug improvement rate	0.55 ± 0.13	0.49 ± 0.15	0.031
UPDRSIII drug off	50.22 ± 13.10	57.71 ± 14.27	0.007
UPDRSIII drug on	22.45 ± 9.29	30.75 ± 11.99	<0.001
NMSS Preop	84.63 ± 30.65	90.38 ± 29.38	0.333
PDQ39 Preop	71.47 ± 17.02	74.98 ± 15.41	0.275
MOCA Preop	23.76 ± 3.44	13.67 ± 3.57	<0.001
MMSE Preop	26.88 ± 3.24	23.13 ± 3.25	<0.001
HAMD Preop	14.71 ± 5.11	16.73 ± 7.82	0.124
HAMA Preop	17.47 ± 5.31	18.63 ± 5.45	0.275
Gender			0.465
male	33 (64.71%)	30 (57.69%)	
female	18 (35.29%)	22 (42.31%)	
H-Y			0.659
2	1 (1.96%)	1 (1.92%)	
2.5	9 (17.65%)	7 (13.46%)	
3	27 (52.94%)	23 (44.23%)	
4	12 (23.53%)	16 (30.77%)	
5	2 (3.92%)	5 (9.62%)	

**Table 2:** Effect of characteristics of patients on improvement rate of cognitive state.

	Statistics	OR (95% CI) p-Value
Age	59.01 ± 8.03	1.06 (1.00, 1.11) 0.0362
Gender		
male	63 (61.17%)	1.0
female	40 (38.83%)	1.34 (0.61, 2.98) 0.4657
Education (years)	5.54 ± 4.38	1.42 (1.23, 1.63) <0.0001
LED	659.95 ± 363.80	1.00 (1.00, 1.00) 0.9761
UPDRSIII drug off	54.00 ± 14.15	1.04 (1.01, 1.07) 0.0090
UPDRSIII drug on	26.64 ± 11.47	1.08 (1.03, 1.12) 0.0005
Drug improvement rate	0.52 ± 0.14	0.04 (0.00, 0.79) 0.0339
MMSE Preop	24.99 ± 3.74	0.70 (0.61, 0.82) <0.0001
MOCA Preop	18.67 ± 6.15	0.46 (0.34, 0.64) <0.0001
NMSS Preop	87.53 ± 30.01	1.01 (0.99, 1.02) 0.3303
PDQ39 Preop	73.24 ± 16.25	1.01 (0.99, 1.04) 0.2737
HAMD Preop	15.73 ± 6.66	1.05 (0.99, 1.12) 0.1292
HAMA Preop	18.06 ± 5.38	1.04 (0.97, 1.12) 0.2726
Duration (years)	8.70 ± 3.80	0.98 (0.88, 1.08) 0.6637
H-Y		
2	2 (1.94%)	1.0
2.5	16 (15.53%)	0.78 (0.04, 14.75) 0.8671
3	50 (48.54%)	0.85 (0.05, 14.39) 0.9115
4	28 (27.18%)	1.33 (0.08, 23.54) 0.8443
5	7 (6.80%)	2.50 (0.10, 62.61) 0.5771

**Table 3:** Multivariate regression showing the effect of education, age, UPDRSIII drug off, UPDRSIII drug on, MoCA Preop, MMSE Preop and drug improvement rate on the improvement rate of cognitive state.

	Non-Adjusted		Model I		Model II	
	OR (95% CI)	p-Value	OR (95% CI)	p-Value	OR (95% CI)	p-Value
Education (years)	1.42 (1.23, 1.63)	***	1.44 (1.23, 1.68)	***	1.49 (1.25, 1.77)	***
Age (years)	1.06 (1.00, 1.11)	*	1.09 (1.01, 1.17)	*	1.07 (0.99, 1.15)	
UPDRSIII drug off	1.04 (1.01, 1.07)	**	1.05 (1.01, 1.09)	**	1.04 (1.00, 1.08)	
UPDRSIII drug on	1.08 (1.03, 1.12)	***	1.09 (1.03, 1.14)	**	1.08 (1.02, 1.14)	**
MoCA Preop	0.46 (0.34, 0.64)	***	0.16 (0.05, 0.54)	**	0.14 (0.04, 0.54)	**
MMSE Preop	0.70 (0.61, 0.82)	***	0.60 (0.48, 0.75)	***	0.61 (0.49, 0.77)	***
Drug improvement rate	0.04 (0.00, 0.79)	*	0.03 (0.00, 0.77)	*	0.04 (0.00, 1.15)	

Model I is adjusted for duration and gender, whereas Model II is adjusted for duration, gender, H-Y, LED, NMSS Preop, PDQ39 Preop. CI, confidence interval; OR, odds ratio. * *p* < 0.05 ** *p* < 0.01 *** *p* < 0.001.

## Data Availability

The original contributions presented in the study are included in the article/Supplementary Materials, further inquiries can be directed to the corresponding author. Informed consent was obtained from all subjects involved in the study.

## Supplementary Materials

The following supporting information can be downloaded at https://www.mdpi.com/article/10.3390/brainsci12060759/s1. Datasheet S1, Dataset used for this investigation.

## References

[1] Cerri S., Mus L., Blandini F. (2019). Parkinson’s Disease in Women and Men: What’s the Difference?. J. Parkinsons Dis..

[2] Ascherio A., Schwarzschild M.A. (2016). The epidemiology of Parkinson’s disease: Risk factors and prevention. Lancet Neurol..

[3] Ribault S., Simon E., Berthiller J., Polo G., Nunes A., Brinzeu A., Mertens P., Danaila T., Thobois S., Laurencin C. (2021). Comparison of clinical outcomes and accuracy of electrode placement between robot-assisted and conventional deep brain stimulation of the subthalamic nucleus: A single-center study. Acta Neurochir..

[4] Pinto S., Le Bas J.F., Castana L., Krack P., Pollak P., Benabid A.L. (2007). Comparison of two techniques to postoperatively localize the electrode contacts used for subthalamic nucleus stimulation. Neurosurgery.

[5] Liu Y., Wu L., Yang C., Xian W., Zheng Y., Zhang C., Hong G., Jiang L., Yang Z., Pei Z. (2020). The white matter hyperintensities within the cholinergic pathways and cognitive performance in patients with Parkinson’s disease after bilateral STN DBS. J. Neurol. Sci..

[6] Farzanehfar P., Woodrow H., Horne M. (2022). Sensor Measurements Can Characterize Fluctuations and Wearing Off in Parkinson’s Disease and Guide Therapy to Improve Motor, Non-motor and Quality of Life Scores. Frony. Aging Neurosci..

[7] Leimbach F., Atkinson-Clement C., Socorro P., Jahanshahi M. (2022). The Effects of Subthalamic Nucleus Deep Brain Stimulation in Parkinson’s Disease on Associative Learning of Verbal and Non-Verbal Information by Trial and Error or with Corrective Feedback. J. Parkinsons Dis..

[8] Wei X., Shen Q., Litvan I., Huang M., Lee R.R., Harrington D.L. (2022). Internetwork Connectivity Predicts Cognitive Decline in Parkinson’s and Is Altered by Genetic Variants. Front. Aging Neurosci..

[9] Bourilhon J., Mullie Y., Olivier C., Cherif S., Belaid H., Grabli D., Czernecki V., Karachi C., Welter M.-L. (2022). Stimulation of the pedunculopontine and cuneiform nuclei for freezing of gait and falls in Parkinson disease: Cross-over single-blinded study and long-term follow-up. Parkinsonism Relat. Disord..

[10] Aarsland D., Bronnick K., Williams-Gray C., Weintraub D., Marder K., Kulisevsky J., Burn D., Barone P., Pagonabarraga J., Allcock L. (2010). Mild cognitive impairment in Parkinson disease: A multicenter pooled analysis. Neurology.

[11] Ding W., Ding L.J., Li F.F., Han Y., Mu L. (2015). Neurodegeneration and cognition in Parkinson’s disease: A review. Eur. Rev. Med. Pharmacol. Sci..

[12] Appleby B.S., Duggan P.S., Regenberg A., Rabins P.V. (2007). Psychiatric and neuropsychiatric adverse events associated with deep brain stimulation: A meta-analysis of 10 years’ experience. Mov. Disord..

[13] Xie Y., Meng X., Xiao J., Zhang J., Zhang J. (2016). Cognitive Changes following Bilateral Deep Brain Stimulation of Subthalamic Nucleus in Parkinson’s Disease: A Meta-Analysis. BioMed Res. Int..

[14] David F.J., Munoz M.J., Corcos D.M. (2020). The effect of STN DBS on modulating brain oscillations: Consequences for motor and cognitive behavior. Exp. Brain Res..

[15] Smeding H.M., Speelman J.D., Huizenga H.M., Schuurman P.R., Schmand B. (2011). Predictors of cognitive and psychosocial outcome after STN DBS in Parkinson’s Disease. J. Neurol. Neurosurg. Psychiatry.

[16] Lövdén M., Fratiglioni L., Glymour M.M., Lindenberger U., Tucker-Drob E.M. (2020). Education and Cognitive Functioning Across the Life Span. Psychol. Sci. Public Interest..

[17] Lyons K., McLaughlin J.E., Khanova J., Roth M.T. (2017). Cognitive apprenticeship in health sciences education: A qualitative review. Adv. Health Sci. Educ. Theory Pract..

[18] McSparron J.I., Vanka A., Smith C.C. (2019). Cognitive learning theory for clinical teaching. Clin. Teach..

[19] Beurrier C., Congar P., Bioulac B., Hammond C. (1999). Subthalamic nucleus neurons switch from single-spike activity to burst-firing mode. J. Neurosc..

[20] Knight E.J., Testini P., Min H.-K., Gibson W.S., Gorny K.R., Favazza C.P., Felmlee J.P., Kim I., Welker K.M., Clayton D.A. (2015). Motor and Nonmotor Circuitry Activation Induced by Subthalamic Nucleus Deep Brain Stimulation in Patients with Parkinson Disease: Intraoperative Functional Magnetic Resonance Imaging for Deep Brain Stimulation. Mayo Clin. Proc..

[21] Wang X.Q., Zhuang H.X., Zhang L.X., Chen X., Niu C.S., Zhao M. (2019). Nomogram for Predicting Postoperative Delirium After Deep Brain Stimulation Surgery for Parkinson’s Disease. World Neurosurg..

[22] Zhan L., Wang X.Q., Zhang L.X. (2020). Nomogram Model for Predicting Risk of Postoperative Delirium After Deep Brain Stimulation Surgery in Patients Older Than 50 Years with Parkinson Disease. World Neurosurg..

[23] Frizon L.A., Hogue O., Achey R., Floden D.P., Nagel S., Machado A.G., Lobel D.A. (2019). Quality of Life Improvement Following Deep Brain Stimulation for Parkinson Disease: Development of a Prognostic Model. Neurosurgery.

